# SUPPORT Tools for evidence-informed health Policymaking (STP) 1: What is evidence-informed policymaking?

**DOI:** 10.1186/1478-4505-7-S1-S1

**Published:** 2009-12-16

**Authors:** Andrew D Oxman, John N Lavis, Simon Lewin, Atle Fretheim

**Affiliations:** 1 Norwegian Knowledge Centre for the Health Services, P.O. Box 7004, St. Olavs plass, N-0130 Oslo, Norway; 2 Centre for Health Economics and Policy Analysis, Department of Clinical Epidemiology and Biostatistics, and Department of Political Science, McMaster University, 1200 Main St. West, HSC-2D3, Hamilton, ON, Canada, L8N 3Z5; 3 Norwegian Knowledge Centre for the Health Services, P.O. Box 7004, St. Olavs plass, N-0130 Oslo, Norway; Health Systems Research Unit, Medical Research Council of South Africa; 4 Norwegian Knowledge Centre for the Health Services, P.O. Box 7004, St. Olavs plass, N-0130 Oslo, Norway; Section for International Health, Institute of General Practice and Community Medicine, Faculty of Medicine, University of Oslo, Norway

## Abstract

This article is part of a series written for people responsible for making decisions about health policies and programmes and for those who support these decision makers.

In this article, we discuss the following three questions: What is evidence? What is the role of research evidence in informing health policy decisions? What is evidence-informed policymaking?

Evidence-informed health policymaking is an approach to policy decisions that aims to ensure that decision making is well-informed by the best available research evidence. It is characterised by the systematic and transparent access to, and appraisal of, evidence as an input into the policymaking process. The overall process of policymaking is not assumed to be systematic and transparent. However, within the overall process of policymaking, systematic processes are used to ensure that relevant research is identified, appraised and used appropriately. These processes are transparent in order to ensure that others can examine what research evidence was used to inform policy decisions, as well as the judgements made about the evidence and its implications. Evidence-informed policymaking helps policymakers gain an understanding of these processes.

## About STP

*This article is part of a series written for people responsible for making decisions about health policies and programmes and for those who support these decision makers. The series is intended to help such people ensure that their decisions are well-informed by the best available research evidence. The SUPPORT tools and the ways in which they can be used are described in more detail in the Introduction to this series *[1].* A glossary for the entire series is attached to each article (see Additional File *1). *Links to Spanish, Portuguese, French and Chinese translations of this series can be found on the SUPPORT website (http://www.support-collaboration.org). Feedback about how to improve the tools in this series is welcome and should be sent to: STP@nokc.no*.

## Scenario

You work in the Ministry of Health and the Minister of Health has asked you to present options for improving the extent to which children are covered by health insurance. You want to ensure that decisions about how to address this important problem are well-informed. You decide to commission a unit that supports the Ministry of Health in using evidence in policymaking to prepare a policy brief summarising both the best available evidence characterising the problem and the options for addressing it.

## Background

For senior policymakers and others involved in scenarios such as the one outlined above, this article provides a basis for a common understanding of what constitutes ‘evidence’, the role of evidence in health policymaking, what constitutes ‘evidence-informed health policymaking’, and why it is important.

The achievement of universal and equitable access to healthcare, of health-related Millennium Development Goals (MDGs), and of other health goals is more likely to be realised through well-informed health policies and actions [2-5]. Unfortunately, the reality is that health policies are often not well-informed by research evidence [5-8]. Poorly-informed decision making is one of the reasons why services sometimes fail to reach those most in need, why health indicators may be off-track and why many countries are unlikely to be able to meet the health MDGs [9]. Poorly-informed decision making may also contribute to problems related to the effectiveness, efficiency (i.e. value for money), and equity of health systems.

Sub-Saharan Africa spends, on average, approximately €80 per person on healthcare. In comparison, Asia spends €190 and OECD high-income countries spend €2,700 per person [10]. With limited resources and a substantial healthcare burden, it is vital that low- and middle-income countries spend their healthcare budgets wisely. High-income countries also face resource constraints due to growing healthcare demands and costs.

Access to health services is often not equitable and this may be exacerbated by inefficient health systems [11]. Once individuals do gain access, care may be substandard or expensive. Effective and cheap interventions, such as magnesium sulphate for eclampsia and pre-eclampsia, are sometimes not used, or are simply unavailable [12]. Ineffective or unnecessarily expensive interventions (such as routine episiotomies, and the provision of intravenous fluids rather than oral rehydration solutions for diarrhoea in children) are sometimes still used. Better use of research evidence for selecting and promoting interventions, and for deciding on the delivery, financial and governance arrangements to support the use of these interventions can help to reduce these problems, as illustrated by the examples shown in Table 1 and Additional File 2.

**Table 1:** Examples of the use of research evidence in policymaking

**Magnesium sulphate for the treatment of eclampsia and pre-eclampsia – an example of inadequate health system arrangements to support an inexpensive and effective intervention** There is high-quality evidence showing that magnesium sulphate, a low-cost drug, is effective for the treatment of eclampsia and pre-eclampsia [31,32]. However, the drug, like many other effective treatments in low- and middle-income countries, is still not yet widely available [12,33]. Failures in the registration, procurement, and distribution mechanisms for magnesium sulphate have contributed to its poor availability in countries such as Mozambique and Zimbabwe [12]. In other countries, problems include a lack of guidelines mandating the use of magnesium sulphate, the failure to include it on lists of essential drugs, a failure to implement existing guidelines, and restrictions on which facilities and health workers are authorised to administer it [33]. Although eclampsia and severe pre-eclampsia affect few women relative to the number of people affected by other healthcare problems, approximately 63,000 women worldwide die from these conditions every year. These conditions are also associated with neonatal deaths. See Additional File 2 for further examples.

An evidence-informed approach better enables policymakers to manage their own use of research evidence. It also enables them to manage better the misuse of research evidence by lobbyists, including researchers when they act as advocates for particular policy positions. Evidence-informed approaches allow policymakers to:

• Ask critical questions about the research evidence available to support advocated policies

• Demonstrate that they are using good information on which to base their decisions, and

• Ensure that evaluations of their initiatives are appropriate and that the outcomes being measured are realistic and agreed in advance 

An evidence-informed approach to policymaking also allows policymakers to acknowledge that policies may be informed by imperfect information. This recognition reduces political risk because it sets in motion ways to alter course if policies do not work as expected. There is a far greater political risk when policies are advocated without acknowledging the limitations of the available evidence and when policies are then adhered to regardless of the results. This renders policymakers subject to criticism for failures related and unrelated to the policy itself.

In this series of articles, our aim is to improve the effectiveness, efficiency and equity of health policies through the better use of research evidence to inform decisions. Our focus is on decisions about how best to organise health systems, including arrangements for delivering, financing and governing health services, and strategies for bringing about change [2,13]. In this series, we use these types of decisions as examples to illustrate the ways in which decision making can be better informed by research evidence. Similar approaches can be used to inform decisions about which programmes, services or drugs are provided [14].

## What is evidence?

Discussions of evidence-based practice and evidence-informed policymaking can generate debate about what exactly constitutes ‘evidence’. A common understanding is that “evidence concerns facts (actual or asserted) intended for use in support of a conclusion” [15]. A fact, in turn, is something known through experience or observation. An important implication of this understanding is that evidence can be used to support a conclusion, but it is not the same as a conclusion. Evidence alone does not make decisions.

This understanding of what evidence is has a number of implications. Firstly, expert opinion is more than just evidence. It is the combination of facts, the interpretation of those facts, and conclusions. Evidence always informs expert opinions. And appropriate use of that evidence requires the identification of those facts (experience or observations) that form the basis of the opinions, as well as an appraisal of the extent to which the facts support the conclusions [16].

Secondly, not all evidence is equally convincing. How convincing evidence is depends on what sorts of observations were made and how well they were made. Research evidence is generally more convincing than haphazard observations because it uses systematic methods to collect and analyse observations. Similarly, well designed and executed research is more convincing than poorly designed and executed research.

Thirdly, judgements about how much confidence can be placed in different types of evidence (in other words, the ‘quality’ of the evidence) are made either implicitly or explicitly. It is better to make these judgements systematically and explicitly in order to prevent errors, resolve disagreements, facilitate critical appraisal, and communicate information. This, in turn, requires explicit decisions about the actual types of evidence that need to be considered.

Fourthly, all evidence is context-sensitive, given that all observations are necessarily context-specific. Judgements therefore always need to be made about the applicability of evidence beyond its original context or setting. It is best to make judgements about the applicability of this evidence systematically and explicitly, for the same reasons that it is best to make judgements about the quality of the evidence in a systematic and explicit way.

Fifthly, ‘global evidence’ – i.e. the best evidence available from around the world – is the best starting point for judgements about the impacts of policies and programmes. Although all evidence is context-sensitive, decisions based on a subset of observations that are presumed to be more directly relevant to a specific context (such as those undertaken in a particular country or population group), can be misleading [17]. Judgements about whether to base a conclusion on a subset of observations are better informed if made in the context of all relevant evidence [18].

Finally, it is necessary that local evidence (from the specific setting in which decisions and actions will be taken) informs most other judgements about problems, options for addressing problems, and implementation strategies. This includes evidence of the presence of modifying factors in specific settings, the degree of need (e.g. the prevalence of disease or risk factors or problems with delivery, financial or governance arrangements), values, costs and the availability of resources.

## What is the role of research evidence in informing health policy decisions?

**Figure 1:**
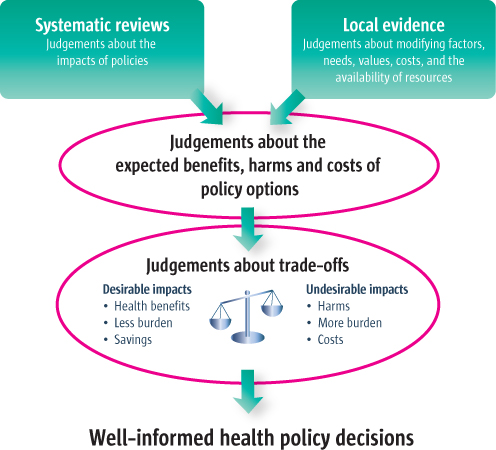
An example of the role of evidence in health policymaking

To make well-informed decisions about issues such as how best to provide universal and equitable access to healthcare, policymakers need access to robust evidence. Evidence is needed to clarify what services and programmes to offer or cover, how to deliver those services, financial arrangements, governance arrangements, and how to implement change [2]. Systematic reviews can be used to inform decisions for key questions within each of these domains [4-6]. An explanation of systematic reviews is provided in Table 2 and examples of systematic reviews are provided in Additional File 3. Figure 1 illustrates the role of evidence from systematic reviews together with local evidence in informing the judgements that need to be made about health policy decisions.

**Table 2:** An explanation of systematic reviews

Systematic reviews are summaries of research evidence that address a clearly formulated question using systematic and explicit methods to identify, select, and critically appraise relevant research, and to collect and analyse data from the studies that are included in the review. Statistical methods (meta-analysis) may or may not be used to analyse and summarise the results of the included studies. Structured summaries of systematic reviews of health system arrangements can be found on the SUPPORT web pages (http://www.support-collaboration.org), including the examples summarised in Additional File 3.

Policy decisions are always influenced by factors other than evidence. These include institutional constraints, interests, ideas (including values), and external factors like recessions. Research evidence is also not the only type of information needed to inform the judgements necessary for policy decision making. Nonetheless, strengthening the use of research evidence, and the ability of policymakers to make appropriate judgements about its relevance and quality, is a critical challenge that holds the promise of helping to achieve significant health gains and better use of resources.

## What is evidence-informed policymaking?

For health policy decision making to be well-informed rather than poorly informed, it is essential that more systematic and transparent processes are applied when accessing and appraising research evidence. Evidence-informed health policymaking is an approach to policy decisions that is intended to ensure that decision making is well-informed by the best available research evidence. How this is done may vary, and will depend on the type of decisions being made and their context. Nonetheless, evidence-informed policymaking is characterised by the fact that its access and appraisal of evidence as an input into the policymaking process is both systematic and transparent. This does not imply that the overall process of policymaking will be systematic and transparent. However, within the overall process of policymaking, systematic processes are used to ensure that relevant research is identified, appraised and used appropriately. These processes are transparent so that others can examine what research evidence has been used to inform policy decisions as well as the judgements made regarding the evidence and its implications.

**Figure 2:**
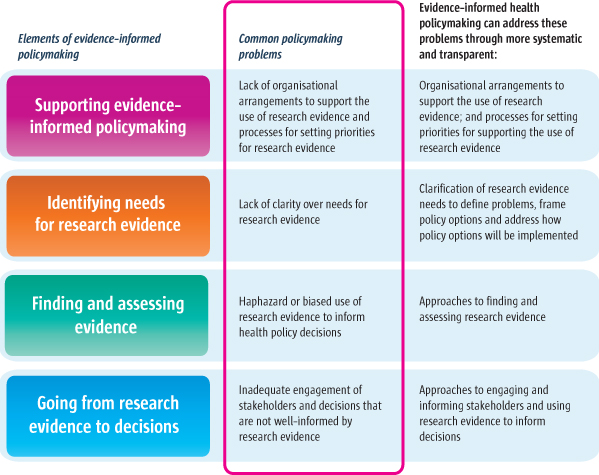
How evidence-informed health policymaking addresses common policymaking problems

In this series, we describe ways in which evidence-informed health policymaking can address common policymaking problems through more systematic and transparent processes to facilitate well-informed decisions, clarify evidence needs, find and assess evidence, and go from evidence to decisions (as illustrated in Figure 2). The advantages of systematic and transparent processes, such as the ones that we describe in this series – compared to processes that are non-systematic and not transparent – are that they can help to protect against errors and bias. This is illustrated by systematic reviews, examples of which are shown in Table 2, which reduce the risk of being misled by chance or by the biased selection and appraisal of evidence.

Different types of evidence are relevant to different questions, and legitimate differences of opinion may exist as to what constitutes the “best available evidence” for particular questions [19]. However, evidence-informed health policymaking aims to ensure that relevant evidence is identified and that judgements about issues such as what evidence is relevant, the reliability and the applicability of identified evidence are made systematically and transparently. Evidence-informed health policymaking also aims to ensure that conflicts of interest do not influence such judgements or any new research that is undertaken in support of policymaking.

Another essential characteristic of evidence-informed policymaking is that policymakers understand the systematic processes used to ensure that relevant research is identified, appraised and used appropriately, as well as the potential uses of such processes. This series of articles is aimed at helping policymakers attain such an understanding.

Since the beginning of the 1990s, there has been a drive towards evidence-based medicine (EBM), which focused initially on decision making by physicians [20,21]. This drive has been extended to other health professionals and consumers, and referred to as ‘evidence-based healthcare’ or ‘evidence-based practice’ as a way of reflecting its broader scope. In the context of management and policymaking, to which this approach has also been extended, it is referred to as “evidence-based policy” [22]. In all of these arenas, debate has focused on what exactly is meant by an evidence-based approach, and how this approach differs from usual practices, as well as the relative benefits and risks. Both EBM and evidence-based policymaking have been criticised for assuming that practice or policy decisions are largely determined by research evidence [4,23-25]. This criticism is largely a misperception of what has been advocated. Neither decisions about individual patients nor policy decisions are determined by evidence alone. Judgements, values, and other factors, always play a role.

Although the terms ‘evidence-based’ and ‘evidence-informed’ can be used interchangeably, we have elected to use the term ‘evidence-informed’ because it better describes the role of evidence in policymaking and the aspiration of improving the extent to which decisions are well-informed by research evidence [4,26].

## What evidence-informed policymaking is not

Like any other tool, those that are used to support the use of evidence to inform policymaking can be misused. Undesirable impacts arising from the inappropriate use of evidence can include inefficient bureaucratic processes, the inappropriate inhibition or delay of promising programmes, the misleading framing of problems, the manipulation of public opinion, and the distortion of the research agenda.Ways in which evidence can be misused include using evidence selectively, stifling the appropriate use of evidence, and creating of a spurious impression of uncertainty. The best way to detect and prevent the inappropriate use of evidence is to use processes that are systematic and transparent, as we will describe in subsequent articles in this series.

## Conclusion

There is growing interest globally in making better use of research evidence in decisions related to health. In 2004, for example, the World Health Organization issued the World Report on Knowledge for Better Health, which included a chapter devoted to linking research to action [27]. The Ministerial Summit on Health Research held that same year in Mexico City, issued a statement on the importance of research for better health and for strengthening health systems [28]. Further, in May 2005, the 58^th^ World Health Assembly passed a resolution acknowledging the Mexico Statement on Health Research, urging member states “to establish or strengthen mechanisms to transfer knowledge in support of evidence-based public health and health-care delivery systems, and evidence-based health-related policies” [29]. The need to continue building on the progress made since the Mexico Ministerial Summit was reflected too in the 2008 Bamako Statement issued by the Ministers of Health, Ministers of Science and Technology, Ministers of Education, and other Ministerial representatives of 53 countries [30]. A first key step towards achieving this objective is to ensure that policymakers and researchers have a shared understanding of what research evidence is and of the role of research evidence in helping to inform policy decisions.

## Resources

### Useful documents and further reading

- Evidence-informed health policy video documentaries: http://www.kunnskapssenteret.no/Artikler/2061.cms – These compelling video documentaries are part of a report on more than 150 organisations, particularly in LMICs, that are building bridges between evidence and policy (http://www.kunnskapssenteret.no/Publikasjoner/469.cms). The video documentaries tell the stories of eight case studies across six continents, where people are trying to improve health systems by using research evidence to inform decision making

- The Mexico statement on health research, 2004 http://www.who.int/rpc/summit/agenda/Mexico_Statement-English.pdf

- World Health Assembly. Resolution on health research, 2005 http://www.who.int/rpc/meetings/58th_WHA_resolution.pdf

- The Bamako call to action on research for health, 2008 http://www.who.int/rpc/news/BAMAKOCALLTOACTIONFinalNov24.pdf

- Chalmers I. If evidence-informed policy works in practice, does it matter if it doesn’t work in theory? Evidence & Policy 2005; 1:227-42 http://www.ingentaconnect.com/content/tpp/ep/2005/00000001/00000002/art00006

- Isaacs D, Fitzgerald D. Seven alternatives to evidence-based medicine. BMJ 1999; 319:1618. http://www.bmj.com/cgi/content/full/319/7225/1618

- Macintyre S, Petticrew M. Good intentions and received wisdom are not enough. Journal of Epidemiology and Community Health 2000; 54:802-3 http://jech.bmj.com/cgi/content/full/54/11/802

- Moynihan R. Using health research in policy and practice: Case studies from nine countries. Milbank Memorial Fund report, 2004 http://www.milbank.org/reports/0409Moynihan/0409Moynihan.html

### Links to websites

- Evidence-Informed Policy Network (EVIPNet): http://www.who.int/rpc/evipnet/en/, http://evipnet.bvsalud.org/php/index.php EVIPNet is an initiative to promote the systematic use of health research evidence in policymaking. Focusing on low- and middle-income countries, EVIPNet promotes partnerships at the country level between policymakers, researchers and civil society in order to facilitate both policy development and policy implementation through the use of the best scientific evidence available.

- Alliance for Health Policy and Systems Research: http://www.who.int/alliance-hpsr/en/ The Alliance HPSR is an international collaboration housed in the World Health Organization (WHO). It aims to promote the generation and use of health policy and systems research as a means to improve the health systems of developing countries.

- Canadian Health Services Research Foundation: http://www.chsrf.ca/home_e.php This Foundation promotes and funds management and policy research in health services and nursing to increase the quality, relevance and usefulness of this research for health system policymakers and managers. In addition, the foundation works with these health system decision makers to support and enhance their use of research evidence when addressing health management and policy challenges.

- UK government’s Policy Hub: http://www.nationalschool.gov.uk/policyhub/index.asp This site aims to promote strategic thinking and improve policymaking and delivery across government. It endeavours to provide users with access to a range of perspectives on policy matters.

## Competing interests

The authors declare that they have no competing interests.

## Authors' contributions

ADO prepared the first draft of this article. JNL, SL and AF contributed to drafting and revising it.

## Supplementary Material

Additional file 1 Glossary

Additional file 2 Further examples of the use of research evidence in policymaking

Additional file 3 Examples of systematic reviews

## References

[B1] Lavis JN Oxman AD Lewin S Fretheim A SUPPORT Tools for evidence-informed health Policymaking (STP). Introduction. Health Res Policy Syst 2009 7 Suppl 1 I1 10.1186/1478-4505-7-S1-I1 20018098 PMC3271819

[B2] Lavis JN Wilson MG Oxman AD Lewin S Fretheim A SUPPORT Tools for evidence-informed health policymaking (STP). 4. Using research evidence to clarify a problem. Res Policy Syst Health Res Policy Syst 2009 7 Suppl 1 S4 10.1186/1478-4505-7-S1-S4 PMC3271831 20018111

[B3] Task Force on Health Systems Research Informed choices for attaining the millennium development goals: towards an international cooperative agenda for health systems research. Lancet 2004 364 997 1003 10.1016/S0140-6736(04)17026-8 15364193

[B4] Chalmers I If evidence-informed policy works in practice, does it matter if it doesn't work in theory? Evidence & Policy 2005 1 2 227 42

[B5] Oxman AD Lavis JN Fretheim A The use of evidence in WHO recommendations. Lancet 2007 369 1883 9 10.1016/S0140-6736(07)60675-8 17493676

[B6] Lavis JN Davies HTO Oxman A Denis JL Golden-Biddle K Ferlie E Towards systematic reviews that inform healthcare management and policymaking. J Health Serv Res Policy 2005 10 35 48 10.1258/1355819054308549 16053582

[B7] Innvaer S Vist G Trommald M Oxman A Health policy-makers' perceptions of their use of evidence: a systematic review. J Health Serv Res Policy 2002 7 239 44 10.1258/135581902320432778 12425783

[B8] Lavis JN Ross SE Hurley JE Hohenadel JM Stoddart GL Woodward CA Abelson J Examining the role of health services research in public policymaking. Milbank Quarterly 2002 80 125 54 10.1111/1468-0009.00005 11933791 PMC2690103

[B9] United Nations The Millennium Development Goals Report. 2007 United Nations http://mdgs.un.org/unsd/mdg/Resources/Static/Products/Progress2007/UNSD_MDG_Report_2007e.pd

[B10] United Nations Development Programme Human Development Report 2006 2006 United Nations Development Programme http://hdr.undp.org/hdr2006/statistics/data

[B11] Victora CG Wagstaff A Schellenberg JA Gwatkin D Claeson M Habicth JP Applying an equity lens to child health and mortality: more of the same is not enough. Lancet 2003 362 233 41 10.1016/S0140-6736(03)13917-7 12885488

[B12] Sevene E Lewin S Marino A Woelk G Oxman A Matinhure S Can a drug be too cheap? The unavailability of magnesium sulphate for the treatment of eclampsia and preeclampsia in Mozambique and Zimbabwe: systems and market failures. BMJ 2005 331 765 16195297 10.1136/bmj.331.7519.765 PMC1239984

[B13] Lavis JN Posada FB Haines A Osei E Use of research to inform public policymaking. Lancet 2004 364 1615 21 10.1016/S0140-6736(04)17317-0 15519634

[B14] Guyatt G Rennie D Meade MO Cook DJ Users' Guides to the Medical Literature. A Manual for Evidence-Based Clinical Practice 2008 Second McGraw Hill

[B15] Lomas J Culver T McCutcheon C McAuley L Law S Conceptualizing and Combining evidence for health system guidance. 2005 Canadian Health Services Research Foundation http://www.chsrf.ca/other_documents/evidence_e.php

[B16] Schuneman HJ Fretheim A Oxman A Improving the Use of Research Evidence in Guideline Development: 9. Grading evidence and recommendations. Health Res Policy Syst 2006 4 12 17147810 10.1186/1478-4505-4-12 PMC1697807

[B17] Counsell CE Clarke MJ Slattery J Sandercock PA The miracle of DICE therapy for acute stroke: fact or fictional product of subgroup analysis? BMJ 1994 309 1677 81 7819982 10.1136/bmj.309.6970.1677 PMC2542663

[B18] Guyatt G Wyer P Ioannidis J Guyatt G, Rennie D, Meade MO, Cook DJ When to believe a subgroup analysis. Users' Guide to the Medical Literature. A Manual for Evidence-Based Clinical Practice 2008 New York: McGraw Hill 571 93

[B19] Oxman AD Fretheim A Schuneman HJ Improving the Use of Research Evidence in Guideline Development: 7. Deciding what evidence to include. Health Res Policy Syst 2006 4 19 17140445 10.1186/1478-4505-4-19 PMC1702350

[B20] Evidence-Based Medicine Working Group Evidence-based medicine. A new approach to teaching the practice of medicine. JAMA 1992 268 2420 5 10.1001/jama.268.17.2420 1404801

[B21] Oxman AD Sacket DL Guyatt G for the Evidence-Based Medicine Working Group Users' guides to the medical literature, I. How to get started. JAMA 1993 270 2093 5 10.1001/jama.270.17.2093 8411577

[B22] Packwood A Evidence-based policy: rhetoric and reality. Social Policy and Society 2002 1 3 267 72

[B23] Straus SE McAlister FA Evidence-based medicine: a commentary on common criticisms. CMAJ 2000 163 837 41 11033714 PMC80509

[B24] Clarence E Technocracy reinvented: the new evidence based policy movement. Public Policy and Administration 2002 17 1 11 10.1177/095207670201700301

[B25] Parsons W From muddling through to muddling up – evidence based policy making and the modernisation of British Government. Public Policy and Administration 2002 17 43 60 10.1177/095207670201700304

[B26] Nutley S Bridging the policy/research divide: reflections and lessons from the UK. 2003 St. Andrews: University of St. Andrews http://www.treasury.govt.nz/publications/media-speeches/guestlectures/pdfs/tgls-nutley.pdf

[B27] World Health Organization World report on knowledge for better health. 2004 World Health Organisation http://www.who.int/rpc/wr2004

[B28] World Health Organization The Mexico statement on health research. 2004 World Health Organization http://www.who.int/rpc/summit/agenda/Mexico_Statement-English.pdf

[B29] World Health Assembly Resolution on health research. 2005 World Health Organization http://www.who.int/rpc/meetings/58th_WHA_resolution.pdf

[B30] World Health Organization The Bamako call to action on research for health. 2008 World Health Organization http://www.who.int/gb/ebwha/pdf_files/EB124/B124_12Add2-en.pdf

[B31] Duley L Henderson-Smart D Magnesium sulphate versus diazepam for eclampsia. Cochrane Database Syst Rev 2003 4 CD000127 14583910 10.1002/14651858.CD000127

[B32] Duley L Gulmezoglu AM Henderson-Smart DJ Magnesium sulphate and other anticonvulsants for women with pre-eclampsia. Cochrane Database Syst Rev 2003 2 CD000025 12804383 10.1002/14651858.CD000025

[B33] Langer A Villar J Tell K Kim T Kennedy S Reducing eclampsia-related deaths--a call to action. Lancet 2008 371 705 6 10.1016/S0140-6736(08)60321-9 18313488

