# Supplementary material for: SUPPORT Tools for evidence-informed health Policymaking (STP) 1: What is evidence-informed policymaking?
Source: Health Res Policy Syst. 2009 Dec 16;7(Suppl 1):S1. doi: 10.1186/1478-4505-7-S1-S1 (PMC3271820; doi:10.1186/1478-4505-7-S1-S1)
Supplement: Additional file 2 — Further examples of the use of research evidence in policymaking [file 1478-4505-7-S1-S1-S2.doc]

**Additional File 2. Further examples of the use of research evidence in policymaking**

**Paying for performance – an example of the widespread use of a health system arrangement with uncertain effects and inadequate impact evaluation**

Paying for performance (P4P) refers to the transference of money or material goods conditional on people taking a measurable action or achieving a predetermined performance target. P4P is widely advocated and used with the aim of improving healthcare quality and utilisation, and achieving other health goals, including the MDGs. An overview of the effects of any type of P4P in the health sector targeted at patients, providers, organisations or governments found 12 systematic reviews [1]. The results indicated that financial incentives targeting recipients of healthcare and individual healthcare professionals appear to be effective in the short run for simple and distinct, well-defined behavioural goals. However, there is limited evidence that financial incentives can sustain long-term changes. There is also limited evidence of the effects of P4P targeted at organisations, or of the effects of P4P in LMICs. In LMICs, P4P schemes have generally included ancillary components, such as increased resources, training and technical support. Evaluations of these schemes have rarely assessed the effects of conditionality per se. There is almost no evidence of the cost‑effectiveness of P4P. Moreover, P4P can have undesirable effects, including motivating unintended behaviours, distortions (ignoring important tasks that are not rewarded with incentives), gaming (improving or cheating on reporting rather than improving performance), cherry picking (selecting or avoiding patients based on how easy it is to achieve performance targets), the widening of the resource gap between rich and poor, and greater dependence on financial incentives.

**Reference pricing in British Columbia – an example of an evidence-informed approach to more efficient drug policies**

Since 1995, the province of British Columbia (BC) in Canada has operated a Reference Drug Program (RDP) and several related policies have attracted both praise and criticism as strategies for cost containment [2]. The policies were introduced by Pharmacare, the publicly‑funded drug insurance programme operated by the provincial Ministry of Health. Pharmacare had been struggling for years with double‑digit growth in annual drug costs and the aim of the RDP was to provide similar insurance coverage for similar drugs without increasing other health service costs or incurring adverse health events. The RDP was challenged by the pharmaceutical industry who argued that it was hazardous to patients. But the RDP was defended by the Ministry of Health as being evidence-based. The degree to which RDP had achieved its goals was evaluated by independent researchers, and this provided the basis for the Ministry of Health to defend and sustain the programme. Researchers needed to adapt to the policymakers’ context, which included competing definitions of medical necessity and a policy cycle that accelerated and decelerated rapidly [3-6]. The sustained involvement of researchers in an advisory committee on policy implementation built mutual respect and understanding between researchers and policymakers, and the smooth implementation of a randomised policy trial. However, the personal collaborative relationships established between the policymakers and researchers were not easily transferable to new staff who did not share the history.

**Seguro Popular in Mexico – an example of an evidence-informed approach to extending health insurance coverage and evaluating its impacts**

In 2004, Mexico’s national government rolled out a new system of health insurance called the Seguro Popular, or the Popular Health Insurance scheme, with the aim of extending coverage to the approximately 50 million Mexicans not covered by existing programmes [7-9]. The scheme was progressively introduced across Mexico, starting with the poorest communities first, and offered a defined package of health services. According to Julio Frenk, Mexico’s Secretary of Health during this time: “This is almost a textbook case of how evidence really first of all changed public perceptions, then informed the debate, and then got translated into legislation” [7]. One of the key pieces of initial evidence that sparked widespread debate about the need for reform was the finding that Mexico’s old health system, contrary to popular belief, was funded largely regressively through private out-of-pocket contributions. Having informed the debate and the development of the scheme, evidence has also played a role in evaluation. Taking advantage of the timetable of the progressive rollout, the government set up a controlled trial that compared the outcomes for those communities receiving the scheme, and those still waiting for it. In Mexico, evidence that flows from evaluative research, such as the controlled study of the Seguro Popular, is seen as central to the nation’s reinvigorated democracy. In 2004, recognising its political and ethical obligation to evaluate the impact of policy decisions, the government of Mexico passed legislation requiring that impact evaluations be conducted for a variety of public programmes, explicitly recognising the value of learning what works – and why – as a guide for future budget decisions [10, 11].

**References**

1. Oxman AD, Fretheim A: **paying for results help to achieve the Millennium Development Goals? Overview of the effectiveness of results-based financing**. Journal of Evidence-Based Medicine 2009; 2:70-83.

2. Fox DM, Oxman A: *Informing Judgment: Case Studies of Health Policy and Research in Six Countries*. New York: Milbank Memorial Fund; 2001.

3. Maclure M, Carleton B, Schneeweiss S: **Designed delays versus rigorous pragmatic trials: lower carat gold standards can produce relevant drug evaluations.** *Med Care* 2007, **45:**S44-9.

4. Schneeweiss S, Patrick AR, Sturmer T, Brookhart MA, Avorn J, Maclure M, Rothman KJ, Glynn RJ: **Increasing levels of restriction in pharmacoepidemiologic database studies of elderly and comparison with randomized trial results.** *Med Care* 2007, **45:**S131-42.

5. Maclure M, Nguyen A, Carney G, Dormuth C, Roelants H, Ho K, Schneeweiss S: **Measuring prescribing improvements in pragmatic trials of educational tools for general practitioners.** *Basic Clin Pharmacol Toxicol* 2006, **98:**243-52.

6. Schneeweiss S, Maclure M, Carleton B, Glynn RJ, Avorn J: **Clinical and economic consequences of a reimbursement restriction of nebulised respiratory therapy in adults: direct comparison of randomised and observational evaluations.** *BMJ* 2004, **328:**560.

7. Moynihan R, Oxman A, Lavis JN, Paulsen E: *Evidence-Informed Health Policy: Using Research to Make Health Systems Healthier.* Rapport nr. 1-2008. Oslo: Nasjonalt kunnskapssenter for helsetjenesten; 2008.

8. Frenk J, Gonzalez-Pier E, Gomez-Dantes O, Lezana MA, Knaul FM: **Comprehensive reform to improve health system performance in Mexico.** *Lancet* 2006, **368:**1524-34.

9. Frenk J: **Bridging the divide: global lessons from evidence-based health policy in Mexico.** *Lancet* 2006, **368:**954-61.

10. Savedoff WD, Levine R, Birdsall N: *When will we ever learn? Improving lives through impact evaluation*. Washington DC: Center for Global Development; 2006.

11. Congress of the United Mexican States.General Law of Social Development: *Title V. Evaluation of the Social Development Policy. [Ley General de Desarrollo Social. Publicado en el Diario Oficial de la Federación 20 de enero de 2004. Título quinto "De la Evaluación de la Política de Desarrollo Social".].* New Law DOF 20-01-2004. Mexico City: Center for Documentation, Information and Analysis; 2004.
