# Supplementary material for: SUPPORT Tools for evidence-informed health Policymaking (STP) 1: What is evidence-informed policymaking?
Source: Health Res Policy Syst. 2009 Dec 16;7(Suppl 1):S1. doi: 10.1186/1478-4505-7-S1-S1 (PMC3271820; doi:10.1186/1478-4505-7-S1-S1)
Supplement: Additional file 3 — Examples of systematic reviews [file 1478-4505-7-S1-S1-S3.doc]

**Additional File 3. Examples of systematic reviews**

**Lay health workers in primary care for maternal and child health – an example of a delivery arrangement [1]**

Lay health workers have no formal professional education, but they are usually provided with job-related training. They can be involved in either paid or voluntary care. They perform diverse functions related to healthcare delivery and a range of terms is used to describe them, including village health workers, community volunteers and peer counsellors, among others. A systematic search for randomised trials of lay health workers up to August 2006 found 48 trials relevant to maternal and child health and high burden diseases. There was variation in the recruitment and training methods, targeted recipients, settings, and the outcomes that were measured. Key findings included:

- The use of lay health workers in maternal and child health programmes shows promising benefits compared to usual care or no intervention in:
- Increasing the uptake of immunisation in children
- Promoting breastfeeding
- Reducing mortality in children under five years, and
- Reducing morbidity from common childhood illnesses
- Little evidence is available regarding the effectiveness of substituting health professionals with lay health workers or the effectiveness of alternative strategies for training, supporting and sustaining lay health workers.

**Direct patient payments for drugs – an example of a financial arrangement [2]**

Policies in which consumers pay directly for their drugs when they fill a prescription include *caps* (the maximum number of prescriptions or drugs that is reimbursed), *fixed co-payments* (people pay a fixed amount per prescription or drug), *tier co-payments* (people pay a fixed amount per prescription or drug, the cost of which may depend, for example, on whether the prescription is for a brand (patented) drug or a generic), *coinsurance* (people pay a percent of the price of the drug), and *ceilings* (for example, people pay either part of the price or the full price of a drug up to a set maximum amount over a year, and thereafter either no – or less – money). A systematic search for studies that evaluated the impacts of these policies found 21 studies reporting on 30 highly varied interventions. Key findings included:

- Cap, coinsurance with a ceiling, and co-payment polices can reduce drug use and save expenditures for drug policies or health plans
- Reductions in drug use were found for life-sustaining drugs and drugs that are important in treating chronic conditions, as well as for other drugs
- Although insufficient data on health outcomes were available, large decreases in the use of drugs that are important for people’s health may have adverse effects. This could lead to the increased use of healthcare services and therefore to increases in overall spending
- Policies in which people pay directly for their drugs are less likely to cause harm only if non-essential drugs are included in these policies, or if exemptions are built into the policies to ensure that people receive needed medical care.

**Consumer involvement – an example of a governance arrangement [3]**

The importance of consumer involvement in healthcare is widely recognised, but a systematic review found that there is extremely sparse evidence about how best to do this for health policymaking. Consumers can be involved in developing healthcare policy through consultations to elicit their views or through collaborative processes. Consultations can be single events or repeated events, large- or small-scale. They can involve individuals or groups of consumers in order to allow debate. The groups may be convened especially for the consultation or they may be established consumer organisations. They can be organised in different forums and through different media. A systematic search up to May 2006 for studies that compared the effects of different methods of involving consumers found only one small study of methods designed to involve consumers in health policymaking. This study provided very low-quality evidence that telephone discussions and face-to-face group meetings engaged consumers better than mailed surveys for setting priorities for community health goals, and resulted in different priorities being set.

**Continuing education meetings – an example of a strategy to bring about change [4]**

Educational meetings (lectures, workshops and courses) are one of the most common types of continuing education for health professionals. The meetings can be highly variable in terms of content, number of participants, the degree and type of interaction, as well as length and frequency. A systematic search for randomised trials of audit and feedback up to March 2006 found 81 studies that met the inclusion criteria for the review. In most of the trials the participants were physicians. The interventions varied with respect to their content and format, and there was wide variation in outcome measures. Key findings included:

- Educational meetings can improve professional practice and healthcare outcomes for the patients
- The median effect is small to modest and comparable to the effect of other continuing medical education activities such as audit and feedback and educational outreach visits
- There are large variations in the effects found in different studies and no firm conclusions can be drawn about what is the most effective form
- The effect appears to be larger with educational meetings that include both interactive and didactic components

**References**

1. Lewin S, Munabi-Babigumira S, Bosch-Capblanch X, Aja G, van Wyk B, Glenton C, Scheel IB, Zwarenstein M, Daniels K: *Lay health workers in primary and community health care: A systematic review of trials*. Geneva: World Health Organization; 2006.

2. Austvoll-Dahlgren A, Aaserud M, Vist G, Ramsay C, Oxman AD, Sturm H, Kosters JP, Vernby A: **Pharmaceutical policies: effects of cap and co-payment on rational drug use.** *Cochrane Database Syst Rev* 2008, 1:CD007017.

3. Nilsen ES, Myrhaug HT, Johansen M, Oliver S, Oxman AD: **Methods of consumer involvement in developing healthcare policy and research, clinical practice guidelines and patient information material.** *Cochrane Database Syst Rev* 2006, 3**:**CD004563.

4. Forsetlund L, Bjorndal A, Rashidian A, Jamtvedt G, O'Brien MA, Wolf F, Davis D, Odgaard-Jensen J, Oxman AD: **Continuing education meetings and workshops: effects on professional practice and health care outcomes.** *Cochrane Database Syst Rev* 2009, 2:CD003030.
